# Sustainable Electrosynthesis of Propylamines through Nitrogen Reduction on a MoS_2_ Catalyst

**DOI:** 10.1021/acselectrochem.5c00490

**Published:** 2026-02-12

**Authors:** Caio V. S. Almeida, Ana. B. Cardile, Lucia H. Mascaro, Frank Marken

**Affiliations:** † Department of Chemistry, Federal University of São Carlos 13565-905 São Carlos, Sao Paulo, Brazil; ‡ Department of Chemistry, 1555 University of Bath, Claverton Down, Bath BA2 7AY, U.K.

**Keywords:** ammonia, electrochemistry, hydrogen, organic amines, catalytic interface

## Abstract

The electrocatalytic utilization of nitrogen compounds for C–N coupling chemistry is a promising research area with significant potential to become a sustainable method for producing organonitrogen molecules. The most commonly employed C–N coupling reaction is reductive amination. In this study, we demonstrate an alternative electrochemical reductive amination reaction using N_2_ gas as the nitrogen source, which enables the production of isopropylamine and diisopropylamine in a single process. For instance, these reactions occur at amorphous MoS_2_-coated carbon paper electrodes. This opens the doors to complex molecule electrosynthesis directly from the gas feed. The effects of the acetone concentration and applied potential on reaction yields are investigated. Direct gas feed is demonstrated as a sustainable electro-organic synthetic technology for organic amines. There is an increase in the production of C_3_H_9_N and C_6_H_15_N with increasing acetone concentration. For 0.4 M acetone under N_2_ (at pH 7.0), isopropylamine and diisopropylamine yield rates are 3.1 and 6.7 μg h^–1^ mg^–1^, respectively, at −0.85 V SCE_sat. KCl_. The highest faradaic efficiency is obtained at −0.75 V SCE_sat. KCl_ for C_3_H_9_N (0.46%) and C_6_H_15_N (2.0%). At more negative potentials, there is a decrease in the production of both amines mainly due to the enhanced/competing HER. In all cases, diisopropylamine is the main organic product. This work provides a new and attractive strategy for organic amine synthesis via one-step electrochemical nitrogen reduction.

## Graphical abstract

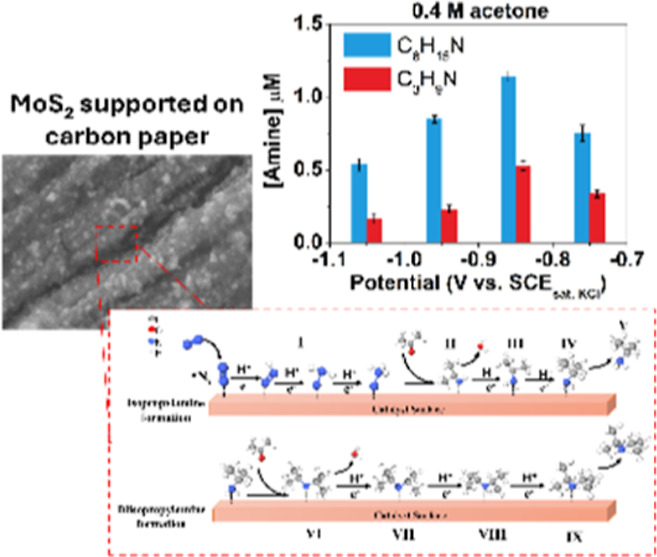

## 1 Introduction

Ammonia is an essential raw material for producing various industrial chemicals, such as fertilizers and pharmaceutical products and is considered an emerging easily transported carrier of hydrogen energy and a carbon-free solar energy storage carrier.^1–3^ However, the mass-produced ammonia requires the Haber–Bosch process, which consumes significant amounts of energy and emits large amounts of greenhouse gases (currently almost 3% of global CO_2_ emissions).^2,3^ Therefore, it is crucial to explore alternative, effective, and sustainable approaches to produce NH_3_ (and related molecules) via N_2_ fixation under much milder process conditions.

The production of NH_3_ can be achieved sustainably using renewable electricity, nitrogen, and water. This can be done indirectly via traditional Haber–Bosch chemistry fed with hydrogen gas produced from water splitting or directly via an electrochemical Haber–Bosch process.^4^ This concern also extends to the additional stages of synthesis in which ammonia is used in the synthesis of chemical compounds. Currently, chemical syntheses involving NH_3_ are multistep processes that operate at high temperatures and pressure.^5–8^ As an alternative, green ammonia can electrochemically react with other renewable resources to form desired nitrogen-containing products.^8^

Aliphatic amines are important chemicals used in the production of dyes, pharmaceuticals, agrochemicals, biologically active compounds, and surfactants.^9,10^ For example, isopropylamine, a low-carbon aliphatic primary amine, is a key intermediate in the production of drugs, herbicides, and pesticides.^9,11^ These chemicals are usually synthesized by the N-alkylation of amines with alkylating reagents, and widely used alkylating reagents include halogenated hydrocarbons, ketones, aldehydes, and alcohols. The N-alkylation of amines by ketones involves two steps: (1) the reaction of ammonia (or an amine) with a carbonyl group to form an imine and (2) the reduction of the imine to form a carbon–nitrogen single bond.^10,12,13^ However, these methods have several drawbacks, such as high cost, low yield, strong corrosion, difficult separation, and an environmentally unfriendly nature. Hence, there is a strong desire for an alternative strategy to produce aliphatic amines under mild conditions.^9,14^

Electrosynthesis stands out as a promising synthetic strategy to enable the sustainable production of chemical feedstocks and added-value compounds by driving reactions under ambient conditions, using electrons generated from renewable electricity instead of redox reagents.^15–17^ Current studies on C–N bond formation mainly focus on the electrochemical NO_3_ reduction intermediates with C_1_–C_2_ molecules (e.g., CO_2_, CO, formic acid, oxalic acid) to produce organonitrogen compounds, including urea, methylamine, and glycine.^18–20^ There are a few recent reports on electrochemical systems, which can build C–N bonds in larger organic molecules (i.e., C_3+_ compounds), such as amino acids and oxime.^15,21,22^ For instance, Rooney and co-workers^22^ developed a one-pot, two-step system for the formation of isopropyl amine. First, a Pd catalyst was employed to convert NO_2_^–^ and acetone into acetone oxime. In the second step, a Pb/PbO catalyst was employed to electroreduce the oxime to amines (with 77% FE at −1.1 V vs. RHE). However, these approaches rely on nitrogen sources such as nitrite or ammonia—rather than directly utilizing molecular nitrogen. The direct electrochemical use of N_2_—especially when sourced from ambient air—remains largely unexplored. This could be a useful process for chemists who want to avoid high-pressure reactions on a large scale with either NH_3_ or H_2_ (i.e., hydrogen gas is sometimes used as the reducing agent).^4^ There is an urgent need to develop cost-effective, high-performance electrocatalysts for achieving energy-efficient processes for the one-pot electrosynthesis of amines.^22^

In this study, we introduce a novel electrochemical pathway and proof of concept for the direct conversion of N_2_ into valuable C–N compounds under ambient conditions (isopropylamine and diisopropylamine). From a conceptual and sustainability perspective, demonstrating C–N bond formation directly from N_2_ represents a more stringent and scientifically significant challenge.

Amorphous MoS_2_ as a catalyst allows for the conversion of nitrogen to amines in a single process. This approach enables the direct conversion of nitrogen and acetone into isopropylamine and diisopropylamine, eliminating the need for prior ammonia generation or molecular hydrogen production. In addition, it extends the scope of MoS_2_-based catalysts for nitrogen electrochemistry reduction beyond ammonia synthesis.

Notably, we find that MoS_2_ catalyst with an optimized acetone concentration (0.4 M) converts N_2_ (pH 7.0) into isopropylamine and diisopropylamine with a yield rate of 3.1 and 6.7 μg h^–1^ mg^–1^, respectively, at −0.85 V SCE_sat. KCl_. Currently, the highest faradaic efficiency is obtained at −0.75 V SCE_sat. KCl_ for C_3_H_9_N (0.46%) and C_6_H_15_N (2.0%). The current work represents a novel strategy for the one-pot electrosynthesis of aliphatic amines from direct N_2_ under ambient conditions, which can upgrade the NRR and enable a more sustainable organic synthesis.

## 2 Experimental Procedure

### 2.1 Reagents

Ammonium tetrathiomolybdate (99.97%), sodium perchlorate (≥98%), monobasic sodium phosphate (≥98%), dibasic sodium phosphate (≥99.0%), *para*-(dimethylamino) benzaldehyde (*p*-C_9_H_11_NO), phenol (≥99%), sodium nitroprusside (≥99.0%), sodium hydroxide (≥97.0%), *N*-(1-Naphtyl) ethylenediamine dihydrochloride (98%), sulfanilamide (98%), sulfamic acid (98%), sodium hypochlorite aqueous solution (6–14% active chlorine), concentrated hydrochloric acid (HCl–37 wt %), sulfuric acid (H_2_SO_4_–98%), nitric acid (HNO_3_–65%), acetone, isopropylamine, and diisopropylamine were obtained from Sigma-Aldrich and used without further purification. A carbon paper electrode (TGP-H-030—total thickness of 110 μm; www.fuelcell.com) was obtained and cut into 0.5 cm × 0.5 cm pieces. Pureshield argon, nitrogen, and oxygen were purchased from BOC, UK. Ultrapure water (18.2 MΩ cm at 20 °C) obtained from a Thermo Scientific water purification system was used to prepare all solutions.

### 2.2 Instrumentation

A potentiostat/galvanostat (Autolab Model GPSTAT12, EcoChemie, The Netherlands) with a three-electrode system was used to carry out all of the electrochemical measurements. The working electrode was MoS_2_/CP, while a graphite rod was used as the counter electrode and a KCl-saturated calomel electrode (SCE) was used as the reference. A 0.1 mol L^–1^ phosphate buffer solution (PBS) at pH 7 was used as the electrolyte solution for all experiments. The MoS_2_/CP catalyst was characterized using a field emission scanning electron microscope (FE-SEM, Jeol JSM-7900F) with an acceleration voltage of 5.0 kV. The ammonia and amine productions were analyzed on reverse phase ion-pairing chromatography coupled to tandem mass spectrometry (Agilent 6545 Accurate-Mass Q-TOF LC/MS system). A Walkup mass spectrometer was used for mass detection with a fragmentor voltage of 80 V and collision energy of 30 V. Peaks were integrated in Mass Hunter (Agilent) software.

### 2.3 Pre-Treatment of Carbon Paper

Before the deposition of the catalysts, the carbon paper substrate needed to be treated in oxidizing acid to increase the hydrophilicity of the material, which is hydrophobic due to the presence of PTFE on its surface. For the acid treatment method, concentrated H_2_SO_4_/HNO_3_ (V/V:3/1) solution was prepared (hazard: mixtures of H_2_SO_4_ and HNO_3_ are highly corrosive). The carbon paper substrate (0.5 cm × 0.5 cm) was dropped into the acid solution and subjected to an ultrasonic agitation bath for 10 min at room temperature. Then, the substrate was rinsed several times with copious amounts of ultrapure water and dried in the oven at 60 °C for 1 h.

### 2.4 Synthesis of MoS_2_ (MoS_2_/CP)

The molybdenum sulfide film was prepared by the electrodeposition technique according to previous reports.^23–26^ The cyclic voltammetry (CV) technique was performed by 50 cycles in the potential range of −1.1 to 0.2 V at 50 mV s^–1^ using 5 mmol L^–1^ (NH_4_)_2_MoS_4_ and 0.1 mol L^–1^ NaClO_4_, previously deaerated with Ar for 15 min.

### 2.5 N_2_ Reduction Reaction (NRR)

The reaction was carried out in 60 mL of 0.1 mol L^–1^ PBS pH 7 in which high-purity N_2_ gas (30 mL min^–1^) was bubbled for 45 min for complete saturation of the medium as well as throughout the entire electrolysis (2 h). The potentials reported in the paper can be converted to the scale of the reversible hydrogen electrode (RHE) with the following equation: *E* (vs. RHE) = *E*_appl_ (vs. SCE _(sat. KCl)_) + pH × 0.059 V + 0.242 V.^24^ Subsequently, the current density (mA cm^–2^_geo_) was recorded with respect to the geometric surface area of 0.5 cm^2^ (counting both front and back of the carbon paper) of the working electrode.

### 2.6 Amine Production

To obtain the amines, different concentrations of acetone (0.05, 0.1, 0.2, 0.4, and 0.8 M) were added to the 0.1 mol L^–1^ PBS electrolyte before the start of the electrolysis, following the same procedure described in Section 2.5.

### 2.7 NH_3_ Detection

The yield amount of NH_3_ (*C*[NH_3_]_N_2__) in the solution was measured by the LC–MS system. Before LC–MS analysis, the solution containing NH_3_ was stained through the indophenol method. Briefly, the following reagent solutions were prepared: S1 was prepared by dissolving 100 mM phenol and 50 mg L^–1^ sodium nitroprusside dihydrate in ultrapure water. S2 was composed of 0.38 M dibasic sodium phosphate, 125 mM sodium hydroxide, and 1% (vol) sodium hypochlorine (10–15% active chlorine). Upon addition of the two reaction solutions (1000 μL each), the samples (200 μL) were mixed and incubated at 37 °C for 40 min. All solutions and reactions were stored at 4 °C until use for LC–MS analysis. A calibration curve for NH_3_ was constructed (Figure S2), using the procedure described above in triplicate, on standard NH_4_Cl solutions prepared in 0.1 mol L^–1^ PBS pH 7 media, with NH_4_^+^ concentrations ranging from 0.00 to 100 μM.

To eliminate the possible exterior sources of contaminations, the corresponding Ar-saturated ( ${{C[NH}_{3}]}_{Ar}$ ) condition and the open-circuit N_2_-saturated condition ( ${{C[NH}_{3}]}_{Open}$ ) for the NRR experiment were used as the baseline for NH_3_ production. Thus, the corrected *C*[NH_3_] produced during the N_2_ reduction was calculated using the following equation (eq 1):

1 $${C[NH}_{3}]={{C[NH}_{3}]}_{N_{2}}-{{C[NH}_{3}]}_{Ar}-{{C[NH}_{3}]}_{Open}$$

The NH_3_ yield rate was determined by (eq 2):

2 $${NH}_{3}\;yield\;rate\;\left(\mu g\;h^{-1}\;{mg}_{cat}^{-1}\right)=({C[NH}_{3}]\times V)/\left(t\times m\right)$$

Here, *C*[NH_3_] is the corrected concentration of NH_3_ production (μg L^–1^); *V* is the volume of the electrolyte (L); *m* is the mass loading of the catalyst on CP (mg); and *t* is the electrolysis reaction time (h). The Faradaic efficiency (FE) can be calculated using the following eq (eq 3):

3 $$FE=\left(3\times F\times {C[NH}_{3}]\times V\right)/17\times Q$$

Here, *F* refers to the Faraday constant (96485.3 C mol^–1^), and *Q* is the quantity of electric charge via the applied potential during the entire experiment (*C*).^27^

### 2.8 N_2_H_4_ Detection

The Watt and Chrisp method was adopted to quantify the N_2_H_4_ in the electrolyte after the reaction.^28^ The coloring agent was prepared by mixing 6.0 g of *p*-C_9_H_11_NO with 30 mL of concentrated HCl and 300 mL of C_2_H_5_OH. Then, 5 mL of the electrolyte was taken from the acid trap and mixed with 5 mL of the coloring agent, followed by stirring for 10 min and standing for 20 min. The absorbance of the solution was constructed using standard hydrazine hydrate solutions ranging from 5 to 35 μM. As demonstrated in Figure S2, a good linear relationship between the absorbance value and the N_2_H_4_ concentration were obtained in three independent calibrations.

### 2.9 NO_*x*_ Detection

The quantification of NO_3_^–^ was measured by following these steps. First, 0.1 mL of electrolyte was mixed with 0.1 mL of 1 M HCl solution and 0.01 mL of 0.8 wt % sulfamic acid solution, and the mixture solution was allowed to sit in the dark for 20 min. The mixed solution was identified by UV–visible spectroscopy (recording the absorption intensity at a wavelength of 210 nm). The calibration curve (Figure S9a,c) was obtained using different concentrations (0.05–2.0 μg mL^–1^) of standard NO_2_^–^ (NaNO_3_, 99%, Sigma-Aldrich) solution.

The concentration of NO_2_^–^ contaminants in the N_2_-saturated 0.1 M PBS electrolyte solution was measured by the Griess-Ilosvay method. Initially, 0.5 g of sulfanilamide was dissolved in 50 mL of a 2.0 mol L^–1^ HCl solution. Then, 20 mg of N-(1- Naphtyl) ethylenediamine dihydrochloride was dissolved in ultrapure water. 0.1 mL of sulfanilamide solution was mixed into 5 mL of electrolyte or standard solution. After 10 min, 0.1 mL of *N*-(-1-Naphthyl) ethylenediamine dihydrochloride solution was added into the above solution. After 30 min, the UV–vis absorption spectrum was measured at a wavelength of 539 cm^–1^. The calibration curve (Figure S9d–f) was obtained using different concentrations (0.01–0.2 μg mL^–1^) of a standard NO_2_^–^ (NaNO_2_, 97%, Sigma-Aldrich) solution.

### 2.10 Amine Detection

The yield amount of isopropylamine and diisopropylamine in the solution was measured by the LC–MS system. Calibration curves for isopropylamine (C_3_H_9_N) and diisopropylamine (C_6_H_15_N) were constructed (Figures S3 and S4, respectively), using standard C_3_H_9_N and C_6_H_15_N solutions prepared in 0.1 mol L^–1^ PBS pH 7 media with concentrations ranging from 0.00 to 10 μM. The amine yield rate was determined by (eq 4):

4 $$amine\;yield\;rate\;\left(\mu g{\;h}^{-1}\;{mg}_{cat}^{-1}\right)=\left(C\left[amine\right]\times V\right)/\left(t\times m\right)$$

Here, (*C*[amine]) is the concentration of isopropylamine and diisopropylamine production (μg L^–1^). The faradaic efficiency (FE) can be calculated by using the following eq (eq 5).

5 FE=(n×F×C[amine]×V)/MW×Q

Here, *n* is the number of electrons involved in isopropylamine (2 electrons) and diisopropylamine (4 electrons) reactions and MW is the molar mass for isopropylamine (59.11 g mol^–1^) and diisopropylamine (101.19 g mol^–1^).

### 2.11 Hydrogen Detection

The hydrogen production amount at different potentials was measured by calibrated gas chromatograph Agilent 7890A equipment.

## 3 Results and Discussion

### 3.1 Electrocatalytic Ammonia Production in the Presence of Acetone

Figure 1a shows the voltammetric profile for the 1st, 10th, 20th, 30th, 40th, and 50th cycle of MoS_2_ electrodeposition onto carbon paper. As the deposition cycles increase, there is an increase in the current densities for the anodic (A) and cathodic (C) reactions, observed at −0.30 V and −0.79 V vs SCE_sat. KCl_, respectively. The gradual increase in the underlying capacitive background is also assigned to coating the surface with amorphous MoS_2_. Further characterization of the amorphous MoS_2_ via SEM, EDS, and XPS was reported.^23,25^ The total charge of the peaks is proportional to the number of deposition cycles, suggesting a continuous deposition of MoS_2_ on the carbon paper.^29^

**Figure 1:**
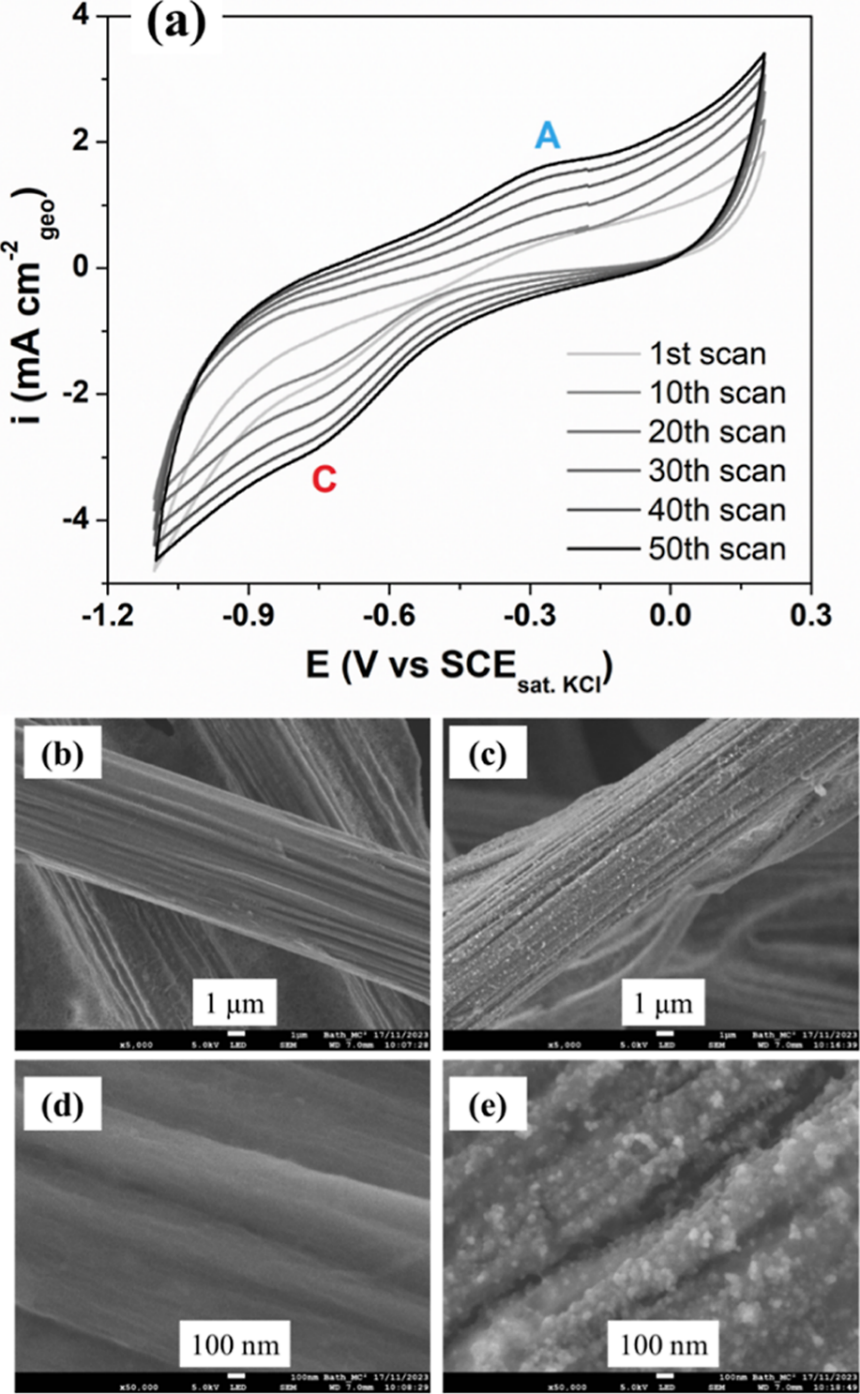
(a) Cyclic voltammograms for 1st, 10th, 20th, 30th, 40th, and 50th cycle of MoS_2_ electrodeposition in a solution containing 5.0 mmol L^–1^ (NH_4_)_2_MoS_4_ in 0.1 mol L^–1^ NaClO_4_ on carbon paper (0.5 cm^2^ for each side of the electrode) with a scan rate of 50 mV s^–1^. SEM images at different magnifications for bare carbon paper: (b) 5k× and (d) 50k× and for MoS_2_/CP: (c) 5k× and (e) 50k×.

The cathodic peak (C) may be related to the reduction of the [MoS4]^2-^ species to MoS_2_ (eq 6).^30^

6 $${[MoS}_{4}{]}^{2-}+{2H}_{2}O+{2e}^{-}\to {MoS}_{2}+{2HS}^{-}+{2OH}^{-}$$

During the anodic sweep, MoS_3_ is formed (A) due to MoS_2_ oxidation in the presence of HS^–^ adsorbed on the surface, as described in eq 7. [MoS_4_]^2–^ may also react with active sites provided by MoS_3_, forming MoS_2_ (eq 3). The reactions ([Disp-formula eq7]) and ([Disp-formula eq8]) continue repeatedly as the number of cycles increases.^30^

7 $${MoS}_{2}+{2HS}^{-}\to {MoS}_{3}+H_{2}S+{2e}^{-}$$

8 $${MoS}_{3}+{[MoS}_{4}{]}^{2-}+{3H}_{2}O+{4e}^{-}\to {2MoS}_{2}{+3HS}^{-}{+3OH}^{-}$$

Figure 1b–e show typical SEM images at different magnifications of the bare carbon paper (Figure 1b–d) and the electrodeposited MoS_2_ (Figure 1c–e). Figure 1e shows a uniform MoS_2_ film covering all of the carbon paper, revealing the spherical form in which the MoS_2_ nuclei grow in the support. This type of morphology is typical for amorphous MoS_2_ films prepared by the electrodeposition method, as demonstrated in the literature.^23,25,31–33^

The XRD analysis and Raman scattering spectra were recorded and are shown in Figure S5a,b to confirm the amorphous nature of the as-prepared catalysts. The XRD pattern of MoS_2_/CP (Figure S5a) does not show the typical peaks for MoS_2_ but instead shows only peaks associated with the hexagonal structure of graphite (PDF No. 00-041-1487) from the carbon paper substrate. These results indicate that the electrodeposited MoS_2_ films are amorphous.^31,33^ Raman spectra (Figure S5b) revealed a poor definition of the characteristic peaks associated with crystalline MoS_2_,^23,31^ which suggests that crystalline MoS_2_ is not present in significant quantities and an amorphous MoS_2_ film was deposited. The broad bands at 375 and 400 cm^–1^ are ascribed to the in-plane Mo–S mode (E_2g_^1^) and the out-plane Mo–S mode (A_1g_), respectively.^34,35^ Raman peaks from 530 to 545 cm^–1^ are assigned to the (S–S)_terminal_ and (S–S)_bridging_ vibrations in disulfide ligands, respectively. In addition, a broad band located at 800–900 cm^–1^ can be ascribed to MoO_3_, indicating the oxidation of MoS_2_ during the synthesis.^34,36^

### 3.2 Effect of Acetone Concentration

The effect of the acetone concentration in the NRR activity of MoS_2_/CP was investigated by linear sweep voltammetry (LSV) measurements. As shown in Figure 2a, the current density in N_2_-saturated solution was greater than that in Ar-saturated solution, demonstrating that MoS_2_ catalyst is active for N_2_ electrochemical reduction.

**Figure 2:**
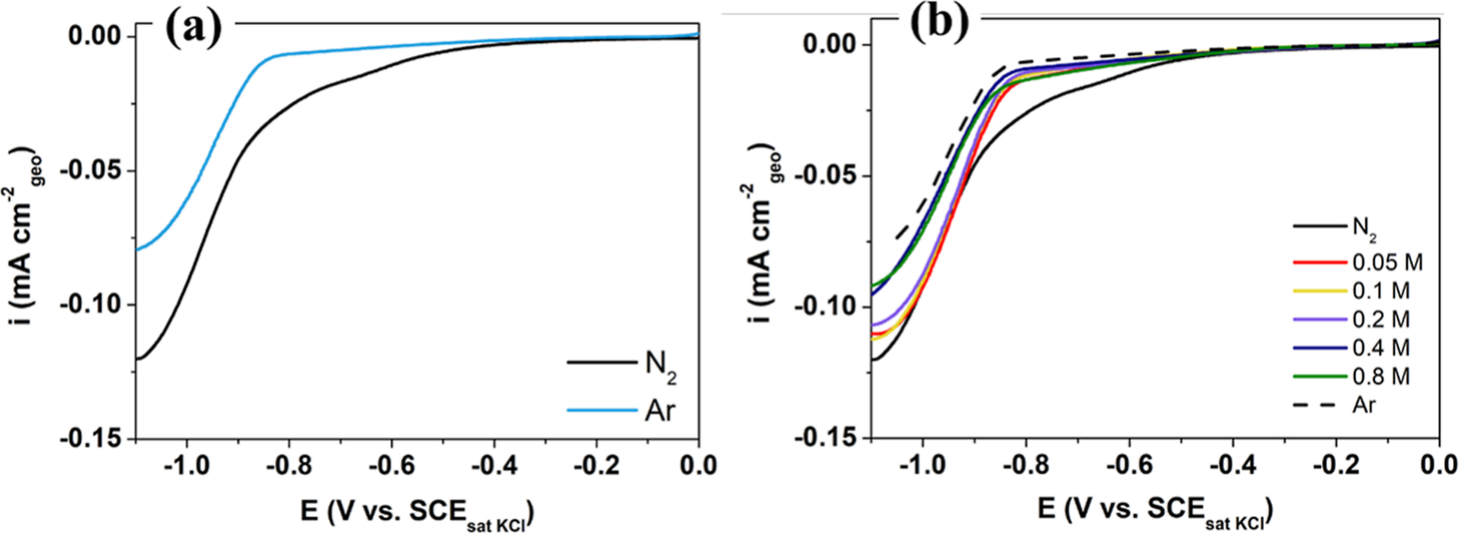
(a) Linear sweep voltammograms (scan rate of 5 mV s^–1^) for MoS_2_/CP in 0.1 mol L^–1^ PBS (pH 7) electrolyte saturated with N_2_ and Ar. (b) Linear sweep voltammograms for MoS_2_/CP with different concentrations of acetone (0.05, 0.1, 0.2, 0.4, and 0.8 M) in 0.1 mol L^–1^ PBS (pH 7) electrolyte saturated with N_2_.

Figure 2b displays LSV curves obtained in N_2_-saturated electrolytes with different acetone concentrations (0.05, 0.1, 0.2, 0.4, and 0.8 M). An increase in acetone concentration results in a decrease in the cathodic current density, particularly in the potential range of −0.5 to −0.9 V. The first assumption is that acetone may be adsorbed on MoS_2_-active sites, hindering the adsorption of N_2_ and consequently reducing the NRR. At higher acetone concentrations (>0.4 M), the cathodic current densities below −0.9 V also decrease, contrary to the trends observed at other acetone concentrations (Figure 2b), suggesting that at high concentrations, acetone also hampers H_2_ adsorption, thereby diminishing the activity of the HER.

Furthermore, the fast increase in the current density starting around −0.90 V in the N_2_-saturated curve could be related to the prevalence of the HER instead of the NRR at higher potentials (or the onset of direct acetone reduction). In the electrolytes containing acetone, this behavior is observed at more positive potentials (∼−0.82 V), which suggests that acetone partially suppresses NRR, facilitating the HER at less negative potentials. To better understand this process, LSV measurements were performed in an Ar-saturated solution at different acetone concentrations (Figure S6). It is possible to observe that there was no significant change in the profile of the curves with an increasing acetone concentration, which supports the idea that the presence of acetone can preferentially block the active sites for N_2_ adsorption.

### 3.3 Electrocatalytic Production of Isopropylamine and Diisopropylamine in the Presence of Acetone

The production of amines during the NRR on the MoS_2_/CP catalyst was assessed further through chronoamperometric tests, followed by LC–MS measurements to quantify isopropylamine and diisopropylamine productions. MoS_2_/CP catalyst was employed in electrolysis for 2 h at −0.85 V vs SCE_sat. KCl_ with different acetone concentrations (Figure 3a) and in 0.4 M acetone at different potentials (Figure 3b). The corresponding counts versus acquisition time for LC–MS data for C_3_H_9_N and C_6_H_15_N are displayed in Figure S7.

**Figure 3:**
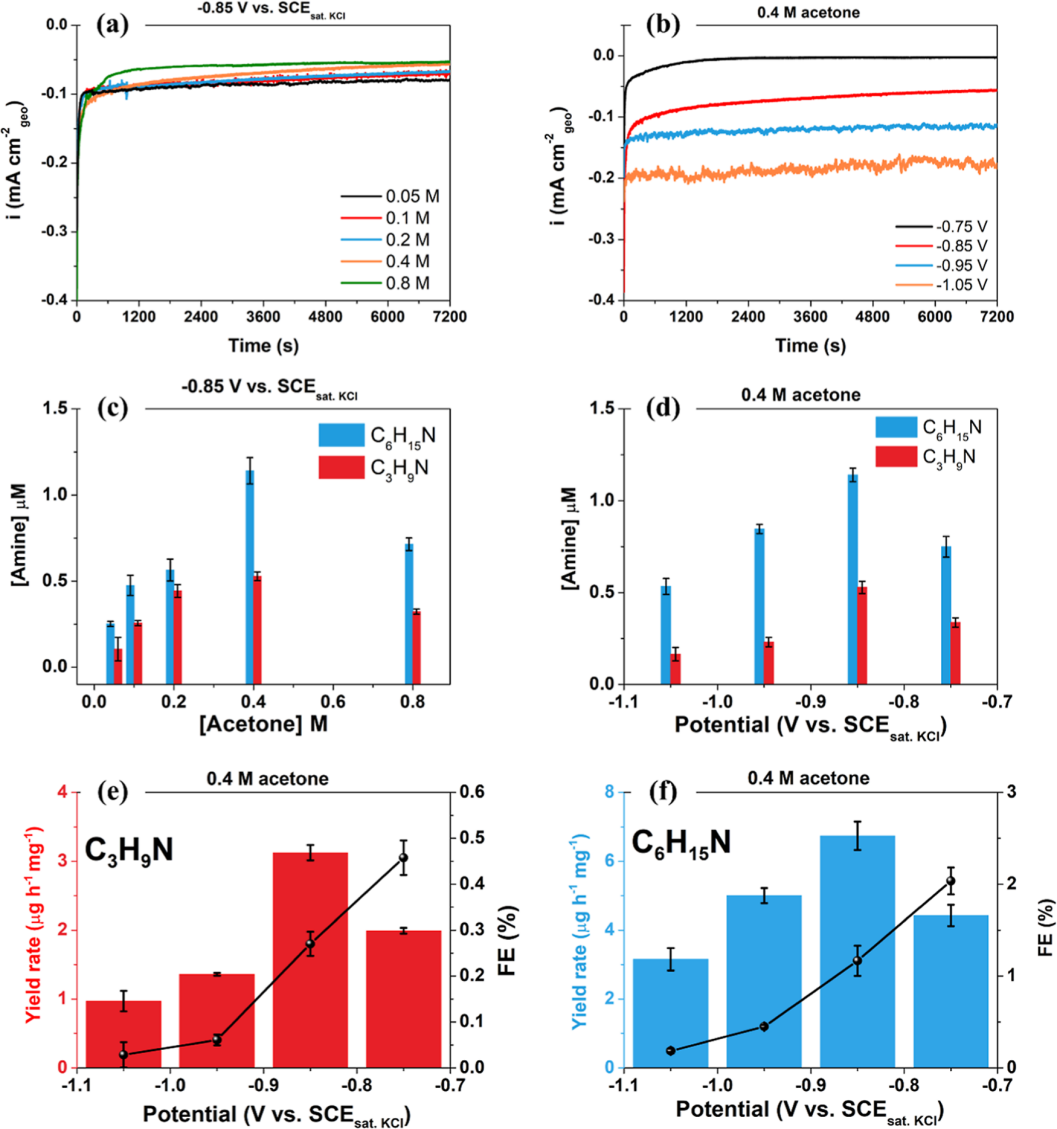
(a) Chronoamperometry data (with stirring, volume 60 mL) in N_2_-saturated 0.1 mol L^–1^ PBS (pH 7) solution (a) at −0.85 V vs SCE_sat. KCl_ with different acetone concentrations and (b) in 0.4 M acetone at different potentials. Isopropylamine (C_3_H_9_N) and diisopropylamine (C_6_H_15_N) production (c) with different acetone concentrations and (d) in 0.4 M acetone at different potentials. Yield rate and FE for (e) isopropylamine (C_3_H_9_N) and (f) diisopropylamine (C_6_H_15_N), employing MoS_2_/CP as the catalyst. Error bars correspond to the standard deviation of triplicate experiments.

As shown in Figure 3c, there is an increase in the production of amines (C_3_H_9_N and C_6_H_15_N) with increasing acetone concentration, reaching a maximum production at 0.4 M acetone of 0.53 and 1.14 μM for C_3_H_9_N and C_6_H_15_N, respectively. For higher acetone concentrations (0.8 M), there is a decrease in the production of C_3_H_9_N and C_6_H_15_N. This decline in production may be attributed to the blocking of MoS_2_ active sites for the NRR and HER reactions due to an excess of acetone, as indicated by the decrease in the cathodic current values during the linear scans (Figure 2b). Analyzing the molar ratio between the production of C_6_H_15_N and C_3_H_9_N (Figure S8a), it is evident that the reaction is more selective for the production of diisopropylamine, regardless of the concentration of acetone used, reaching an average molar ratio of [C_6_H_15_N]/[C_3_H_9_N] of 1.98.

After the best acetone concentration was determined, the effect of the applied potential on the production of amines was investigated using an acetone concentration of 0.4 M. Similar to Figure 3c, there is an increase in the C_3_H_9_N and C_6_H_15_N production when the applied potential is more negative (Figure 3d). The highest C_3_H_9_N and C_6_H_15_N production are achieved at −0.85 V vs. SCE_sat. KCl_. However, when the potential is below −0.85 V vs. SCE_sat. KCl_, the amine production decreases. This decay is attributed here to the enhanced/competing HER at higher overpotentials,^23,37,38^ as confirmed by the calculated FE H_2_ determined by gas chromatography (Figure S9). Combining the FE of H_2_ with the obtained NH_3_, C_3_H_9_N, and C_6_H_15_N selectivity, the unaccounted value of the total FE may be attributed to the capacitance of the carbon support as well as dynamic hydrogen adsorption and absorption on the electrode.^39,40^ Figure S8b shows an increase in the [C_6_H_15_N]/[C_3_H_9_N] molar ratio for potentials above −0.85 V vs. SCE_sat. KCl_, indicating an increase in the selectivity for C_6_H_15_N formation at more negative potentials.

The yield rate and FE for C_3_H_9_N and C_6_H_15_N are shown in Figure 3e,f, respectively. The highest C_3_H_9_N and C_6_H_15_N yield rates of 3.13 and 6.74 μg h^–1^ mg^–1^, respectively, are achieved at −0.85 V SCE_sat. KCl_. However, the higher faradaic efficiency is obtained at −0.75 V SCE_sat. KCl_ for C_3_H_9_N (0.46%) and C_6_H_15_N (2.0%) amines. Below this value, the FE drops drastically, demonstrating the dependence of amine formation on the applied potential. Despite the low FE values, the conversion of nitrogen to amines in a single process was demonstrated.

To achieve further insight into the effect of acetone addition on the NRR activity of MoS_2_, the NRR performance of MoS_2_/CP was compared in the absence (Figure 4a,b) and in the presence of 0.4 M acetone (Figure 4a,b). In the presence of 0.4 M acetone, the catalyst showed an NH_3_ yield rate of 28.5 μg h^–1^ mg^–1^ and an FE of 12.8%, almost a factor of three lower than the NRR in the absence of acetone. As is evident, there is a decrease in the production rates and FE values at all applied potentials, indicating that acetone competes with the adsorption of N_2_ by the active sites of MoS_2_, decreasing the activity for ammonia production. Given the low FE values, hydrogen evolution must be a major competing pathway.

**Figure 4:**
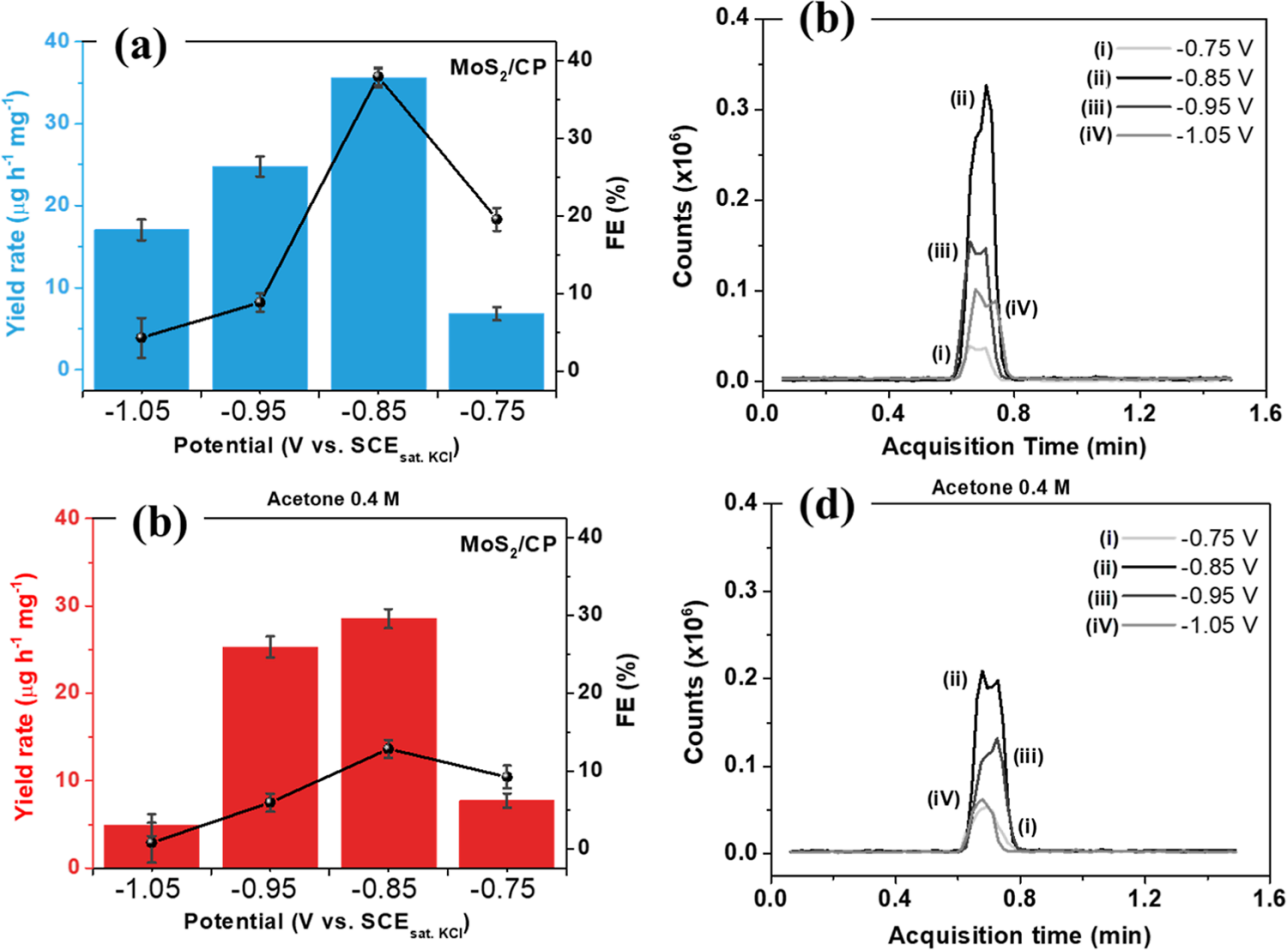
NH_3_ yield rate and FE values for MoS_2_/CP (a) in the absence and (c) in the presence of 0.4 M acetone. Corresponding counts versus acquisition time for LC–MS data for indophenol indicator from ammonia produced at different potentials (b) in the absence and (d) in the presence of 0.4 M acetone. Error bars correspond to the standard deviation of triplicate experiments.

The possible N_2_H_4_ production was detected by the Watt and Chrisp method (Figure S2a,b), but no detectable N_2_H_4_ was observed at different potentials (Figure S2c). The result rules out the formation of hydrazine, indicating that the MoS_2_ catalyst are selective for ammonia production.^20,21^ To confirm the origin of the nitrogen incorporated in the synthesized ammonia, several control experiments were undertaken.^23^ Firstly, the yield of synthesized ammonia was monitored in electrolyte purged with N_2_ and under open circuit conditions (OCP), that is, without current flowing through the cell. This experiment showed a negligible amount of ammonia. Likewise, under applied potential, we did not detect obvious ammonia production unless N_2_ was bubbled in the solution (Figure S10).

Overall, these results evidence the direct correlation between the presence of N_2_ in solution and the generation of ammonia. Additionally, the potential contamination with NO_*x*_ species in the feeding gas was assessed. As can be seen in Figure S11a–f, trace amounts of NO_3_^–^ (Figure S11c) and NO_2_^–^ (Figure S11f) are identified in the feeding N_2_ gas, confirming that NH_3_ was produced by N_2_ reduction in the presence of the MoS_2_ catalyst rather than the sources of contamination.

Stability is another key criterion for evaluating the catalyst performance. To demonstrate the catalyst’s stability, MoS_2_/CP was subjected to consecutive recycling electrolysis at −0.85 V vs. SCE_sat. KCl_ (Figure S12). Figure S12a shows the time-dependent current density curves for MoS_2_/CP in 5 cycles of NRR + 0.4 M acetone. As observed, the current density and the corresponding C_3_H_9_N and C_6_H_15_N yield rates (Figure S12b) in each cycle barely changed. This suggests that the MoS_2_/CP catalyst demonstrates high stability and repeatable NRR performance during the recycling test. After the recycling test, the SEM image (Figure S12c) indicates that the structure and surface morphology of MoS_2_/CP changed minimally after recycling testing. Additionally, an XRD analysis (Figure S12d) confirms that MoS_2_/CP maintains its amorphous structure.

### 3.4 Hypothetical Isopropylamine Production Mechanism

There are some previous reports on the chemical amination reaction mechanism of alcohols or carbonyl compounds to amines.^9,10,40–43^ The electrochemical formation of amines from Schiff base intermediates has been observed in biphasic media.^44^ With ammonia, the production of urea at electrodes has been reported.^45^ Based on the obtained results in this work, the possible reaction mechanism for the amination of acetone during the NRR to isopropylamine and diisopropylamine is suggested in Figure 5. First, N_2_ can be adsorbed and/or reduced on the cathode to form N-containing intermediates, such as *N_2_, *NNH, or NH_3_ (I). Ammonia acts as a nucleophile in chemical reactions, attacking electrophilic carbon centers in ketone to form a C–N bond.^46,47^ According to experimental studies and Density Functional Theory (DFT) analyses,^48–50^ it is more likely that acetone adsorbs on the Mo sites of a MoS_2_ catalyst. As a Lewis base, acetone acts as an electron donor and tends to interact with Lewis acidic sites. These sites are represented by the under-coordinated Mo atoms rather than the chemically inert S-terminated surface.^48^ Therefore, the oxygen atom in acetone strongly binds to the positively charged Mo sites, particularly at the edges of the MoS_2_ structure.^49^

**Figure 5:**
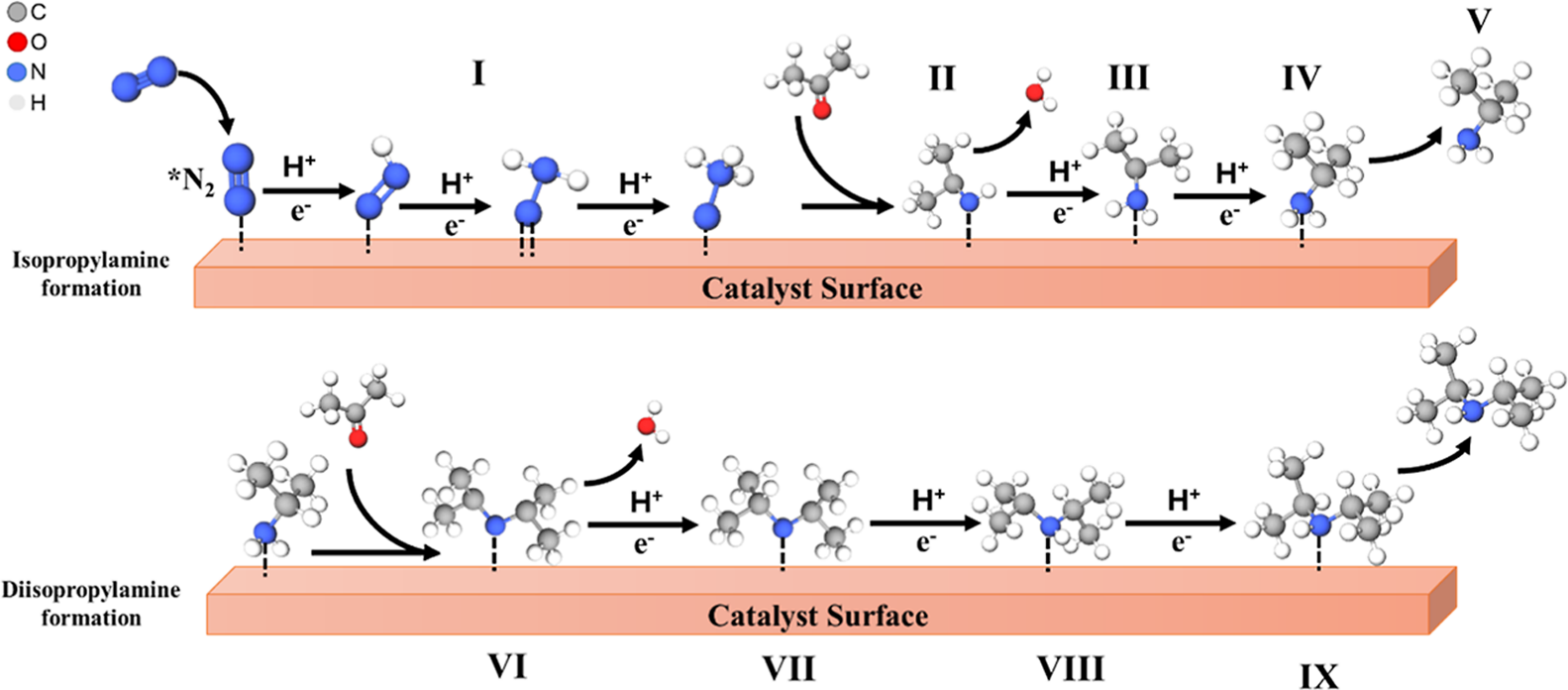
Hypothetical reaction pathway for the catalytic amination of acetone during the NRR to isopropylamine and diisopropylamine over MoS_2_/CP.

Further, acetone reacted with these N-containing species to form the intermediate imine (II). The condensation reaction of acetone with ammonia forms an imine.^9^ It has been reported that the condensation reaction of a ketone with NH_3_ to form an imine is a fast equilibrium reaction.^51^ This intermediate was further hydrogenated (III and IV) to form isopropylamine (V).^9,10^ In addition, isopropanol can form by hydrogenation of acetone with hydrogen under the present reaction conditions.^42^ However, we were unable to detect it by LC–MS, suggesting formation below the analytical detection limit of the equipment.

The formation of diisopropylamine is supposed to occur by the reaction of isopropylamine and acetone (VI), followed by subsequent catalytic hydrogenation reactions (VII to IX).^43^ As demonstrated in Figure 3c,d, diisopropylamine was the main amine produced, and isopropylamine was the subproduct. The variation in acetone concentration showed little influence on the selectivity for amine production. On average, the molar ratio [C_6_H_15_N]/[C_3_H_9_N] remained close to 2:1 during the different concentrations investigated. However, the higher selectivity for diisopropylamine production at more negative potential values indicates that favoring the HER at these potentials allows the hydrogenation of isopropylamine to diisopropylamine. What is indicated in Figure 5 as a surface reaction space could also include interlamellar spaces and defects in amorphous MoS_2_ trapping reaction intermediates.

Finally, we revealed the great benefit of our electrochemical method compared with existing amine synthesis methods. In conventional synthetic chemistry, reductive amination is the most popular methodology for synthesizing amines, which consists of imine formation between aldehydes/ketones and amines followed by imine reduction with a reducing agent. However, chemical reductive amination is mostly conducted in organic solvents because reducing agents can be easily consumed by the hydrogen evolution reaction in aqueous environments. This finding shows that electrochemistry opens up the possibility of expanding the scope of substrates used for reductive amination. We expect our first report of utilizing N_2_ for reductive amination to be exploited for other C–N coupling reactions that require nucleophilic nitrogen species.

## 4 Conclusion and Outlook

In summary, we utilized an amorphous MoS_2_ catalyst that can drive the one-pot electroreduction of dinitrogen in the presence of acetone to amines, with a yield rate of 3.1 and 6.7 μg h^–1^ mg^–1^ for isopropylamine and diisopropylamine, respectively, achieved at −0.85 V SCE_sat. KCl._ in 0.4 M acetone. The highest faradaic efficiency is obtained at −0.75 V SCE_sat. KCl_ for C_3_H_9_N (0.46%) and C_6_H_15_N (2.0%) amines. These values are low but will be improved in future work. Potential strategies to improve faradaic efficiency are (i) modifying the electrode surface morphology, (ii) optimizing the reaction conditions (e.g., pH, supporting electrolyte), and (iii) exploring doping or alternative catalysts. A wider range of amines and related materials might be accessible in situ under the conditions of nitrogen reduction.

This electrochemical method may not only enable the production of important chemical raw materials under mild conditions (room temperature, atmospheric pressure, aqueous solution, neutral pH, no toxic/expensive catalysts) compared to conventional chemical routes but also offers a promising strategy to upgrade nitrogen with sustainable electricity into value-added nitrogen compounds. However, more work is necessary to explore the mechanism, for example, by ^15^N_2_ isotope labeling experiments or by *in operando* spectroelectrochemistry to probe the nature of active catalyst sites or reaction intermediates. Further work will be required to test the catalyst durability and scale-up.

We found that there is an increase in the production of C_3_H_9_N and C_6_H_15_N with increasing acetone concentration, up to 0.4 M. At higher acetone concentrations, the excess of acetone may block MoS_2_ active sites for the NRR and HER reactions, decreasing the level of amine production. However, in all cases, diisopropylamine is the main product during the reaction. The applied potential also plays a role in the production and selectivity of amine. At more negative potentials, there is a decrease in the production of both amines due to enhanced/competing HER at higher overpotentials. In addition, diisopropylamine production is favored in these potentials due to the greater availability of H_2_, facilitating the hydrogenation of isopropylamine to diisopropylamine.

This study presents a new electrochemical approach to enhance nitrogen reduction reaction and support the ecofriendly production of isopropylamine and diisopropylamine. It also broadens the scope of research on electrocatalytic C–N coupling processes for industrial synthesis electrification. In the future, we can further enhance the catalyst performance for increased production and selectivity by improving the electrode composition and morphology, enabling a broader range of processes.

## Supplementary Material



## Notes

Characterization data and supplementary analyses for MoS_2_/CP, including XRD, and Raman results; LC–MS calibration curves for Indophenol Blue, N_2_H_4_, C_3_H_9_N, C_6_H_15_N and UV–VIS calibration curves for NO_3_^–^ and NO_2_^–^; additional linear sweep voltammograms data; and product selectivity figures (PDF)
