# Supplementary material for: Sustainable Electrosynthesis of Propylamines through Nitrogen Reduction on a MoS2 Catalyst
Source: ACS Electrochem. 2026 Feb 12;2(3):705–14. doi: 10.1021/acselectrochem.5c00490 (PMC12969268; doi:10.1021/acselectrochem.5c00490)
Supplement: Supplementary file 1 [file ec5c00490_si_001.pdf]

Supporting Information

Sustainable Electrosynthesis of Propylamines through Nitrogen Reduction on a MoS_2 catalyst

Caio V.S. Almeida,[1,2] Ana B. Cardile,[1,2] Lucia H. Mascaro,*[1] Frank Marken*[2]

[1] *Department of Chemistry, Federal University of São Carlos, Zip Code 13565-905, São Carlos, SP, Brazil*

[2] *Department of Chemistry, University of Bath, Claverton Down, Bath BA2 7AY, UK*

*Corresponding author: fm202@bath.ac.uk (Frank Marken)

*Corresponding author: lmascaro@ufscar.br (Lucia Helena Mascaro)

Content

Figure S1. (a) Photograph of different concentrations of NH_3 stained with the indophenol indicator, (b) corresponding counts versus acquisition time for LC-MS of resultant solutions and (c) calibration of the indophenol blue method for estimating NH_3 concentration, using NH_4Cl solutions of known concentration as standards calibration.

Figure S2. Calibration of the Watt and Chrisp method for estimating N_2H_4 concentration, using N_2H_4 solutions of known concentration as standards. (a) UV-Vis curves of various N_2H_4 concentration after incubated for 30 min at room temperature. The absorbance at 455 nm was measured by UV-Vis spectrophotometer (b) calibration curve used for estimation of N_2H_4 concentration. (c) UV-vis absorption spectra of the electrolyte stained with Watt and Chrisp indicator after NRR electrolysis using MoS_2/CP at -0.85 V vs. $SCE_{sat.\ KCl}$. Error bars correspond to the standard deviation of triplicate experiments.

Figure S3. (a) Calibration curve for estimating isopropylamine (C_3H_9N) concentration, using C_3H_9N solutions of known concentration in 0.1 mol L^{-1} PBS pH 7 media as standards calibration. (b) Corresponding counts versus acquisition time for LC-MS of resultant solutions. Error bars correspond to the standard deviation of triplicate experiments.

Figure S4. (a) Calibration curve for estimating diisopropylamine ($C_6H_{15}N$) concentration, using $C_6H_{15}N$ solutions of known concentration in 0.1 mol L^{-1} PBS pH 7 media as standards calibration. (b) Corresponding counts versus acquisition time for LC-MS of resultant solutions. Error bars correspond to the standard deviation of triplicate experiments.

Figure S5. (a) XRD patterns for MoS_2/CP and (b) Raman spectra (532 nm) recorded on carbon paper and MoS_2/CP materials. Insert: Magnification of the region between 200 and 700 cm^{-1}, highlighting the vibration modes of MoS_2.

Figure S6. Linear sweep voltammograms (scan rate of 5 mV s^{-1}) for MoS_2/CP with different concentrations of acetone (0.05, 0.1, 0.2, 0.4 and 0.8 M) in 0.1 mol L^{-1} PBS (pH 7) electrolyte saturated with Ar.

Figure S7. Corresponding counts versus acquisition time for LC-MS data for C_3H_9N (a, b) and $C_6H_{15}N$ (c, d) with different concentrations of acetone (0.05, 0.1, 0.2, 0.4 and 0.8 M) at –0.85 V vs. $SCE_{sat.\ KCl}$ (a,c) and (b,d) in 0.4 M acetone at different potentials. Identification of (e) isopropylamine (C_3H_9N) and (f) diisopropylamine ($C_6H_{15}N$) by mass spectroscopy (ion source: dual ESI). The peak of isopropylamine and diisopropylamine were identified by searching the possible m/z values of C_3H_9N and $C_6H_{15}N$, respectively, in cation mode, such as (M+H)+, (M+H)+ -H_2O and (M+Na)+.

Figure S8. $[C_6H_{15}N]/[C_3H_9N]$ molar ratio (a) in different concentrations of acetone (0.05, 0.1, 0.2, 0.4 and 0.8 M) at –0.85 V vs. $SCE_{sat.\ KCl}$ and (b) in 0.4 M acetone at different potential applied. These values were taken from Figure 3c and Figure 3d, respectively.

Figure S9. UV-Vis adsorption spectra of known concentration (0.05-2.0 μg mL^{-1}) of NO_3^- in 0.1 M Na_2SO_4. The absorbance at 210 nm was measured by UV-Vis spectrophotometer. (b) The corresponding calibration curve. (c) UV-vis adsorption

spectrum of NO_3^- in N_2-saturated 0.1 mol L^{-1} PBS. (a) UV-Vis adsorption spectra of known concentration (0.05-2.0 μg mL^{-1}) of NO_3^- in 0.1 M PBS. The absorbance at 539 nm was measured by UV-Vis spectrophotometer. (b) The corresponding calibration curve. (c) UV-vis adsorption spectrum of NO_2^- in N_2-saturated 0.1 mol L^{-1} PBS.

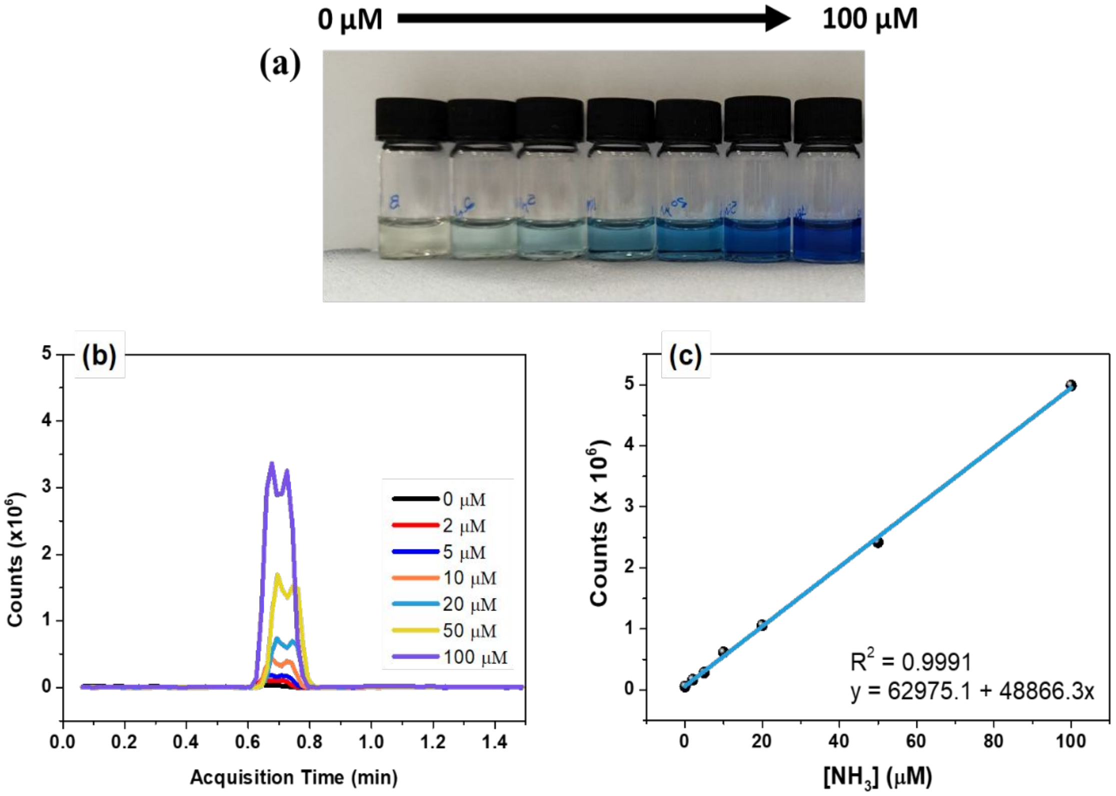

Figure S1. (a) Photograph of different concentrations of NH_3 converted to the indophenol indicator, (b) corresponding counts versus acquisition time for LC-MS of resultant solutions and (c) calibration of the indophenol blue method for estimating NH_3 concentration, using NH_4Cl solutions of known concentration as standards calibration. Error bars correspond to the standard deviation of triplicate experiments.

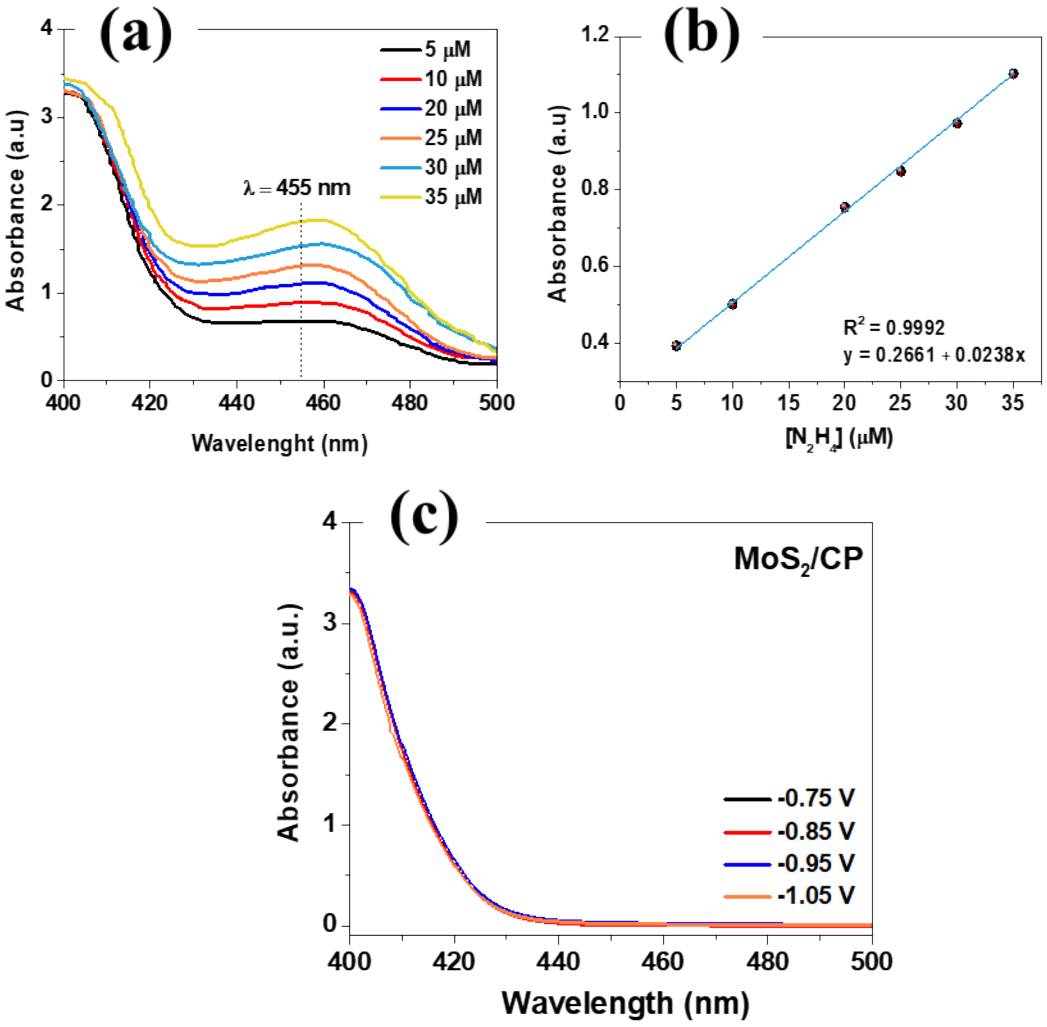

Figure S2. Calibration of the Watt and Chrisp method for estimating N_2H_4 concentration, using N_2H_4 solutions of known concentration as standards. (a) UV-Vis curves of various N_2H_4 concentration after incubated for 30 min at room temperature. The absorbance at 455 nm was measured by UV-Vis spectrophotometer (b) calibration curve used for estimation of N_2H_4 concentration. (c) UV-vis absorption spectra of the electrolyte stained with Watt and Chrisp indicator after NRR electrolysis using MoS_2/CP at -0.85 V vs. $SCE_{sat.\ KCl}$. Error bars correspond to the standard deviation of triplicate experiments.

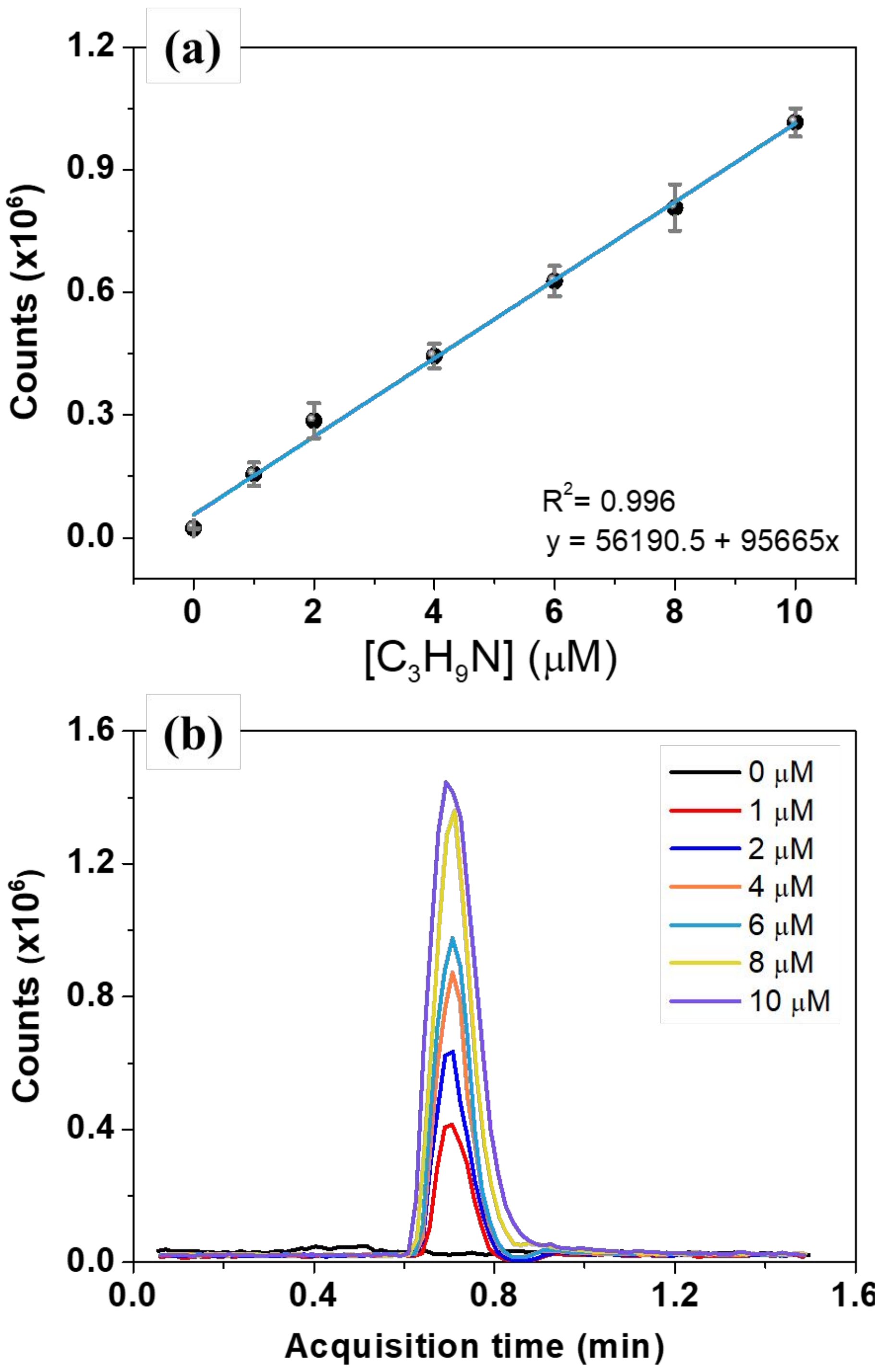

Figure S3. (a) Calibration curve for estimating isopropylamine (C_3H_9N) concentration, using C_3H_9N solutions of known concentration in 0.1 mol L^{-1} PBS pH 7 media as standards calibration. (b) Corresponding counts versus acquisition time for LC-MS of resultant solutions. Error bars correspond to the standard deviation of triplicate experiments.

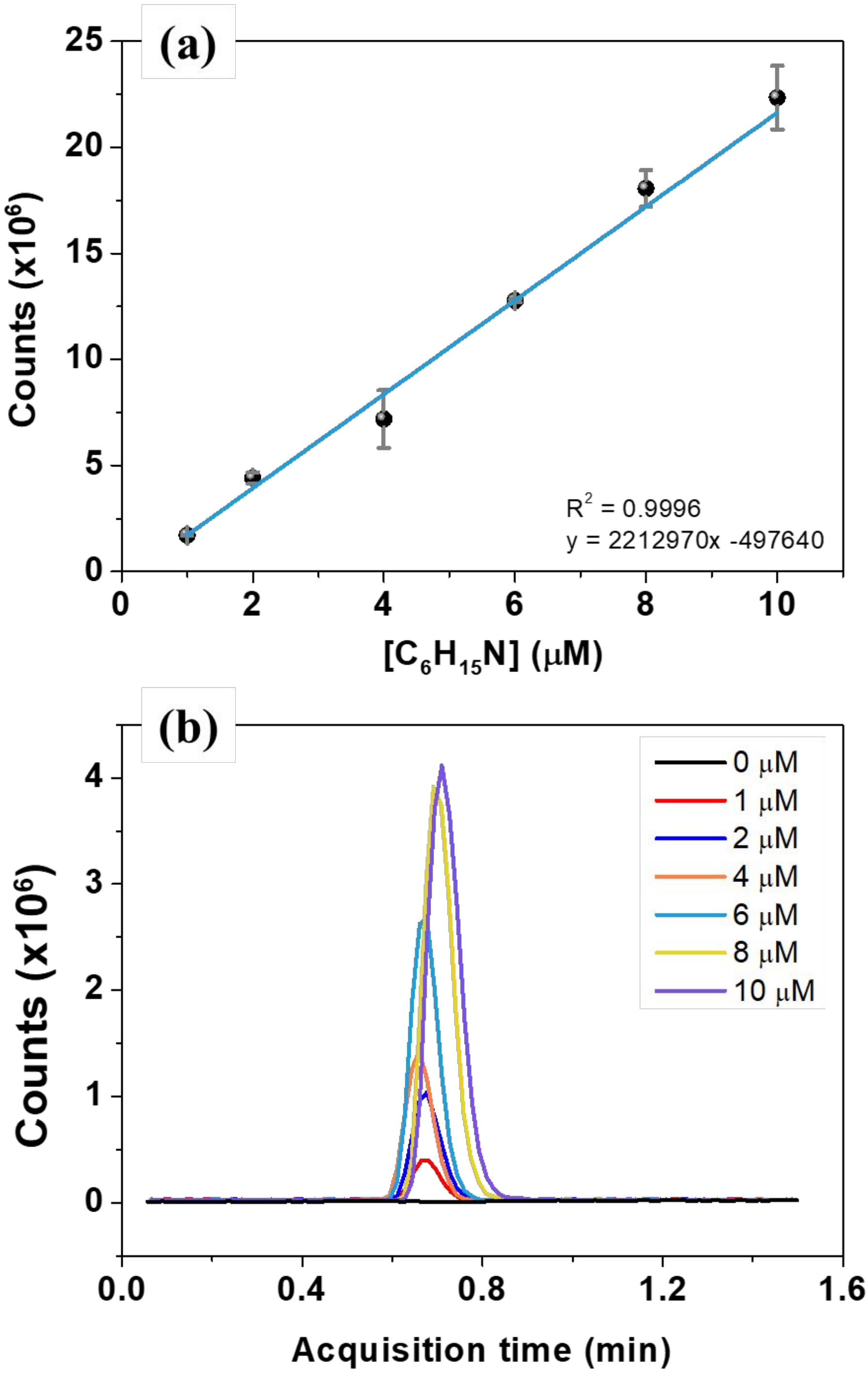

Figure S4. (a) Calibration curve for estimating diisopropylamine ($C_6H_{15}N$) concentration, using $C_6H_{15}N$ solutions of known concentration in 0.1 mol L^{-1} PBS pH 7 media as standards calibration. (b) Corresponding counts versus acquisition time for LC-MS of resultant solutions. Error bars correspond to the standard deviation of triplicate experiments.

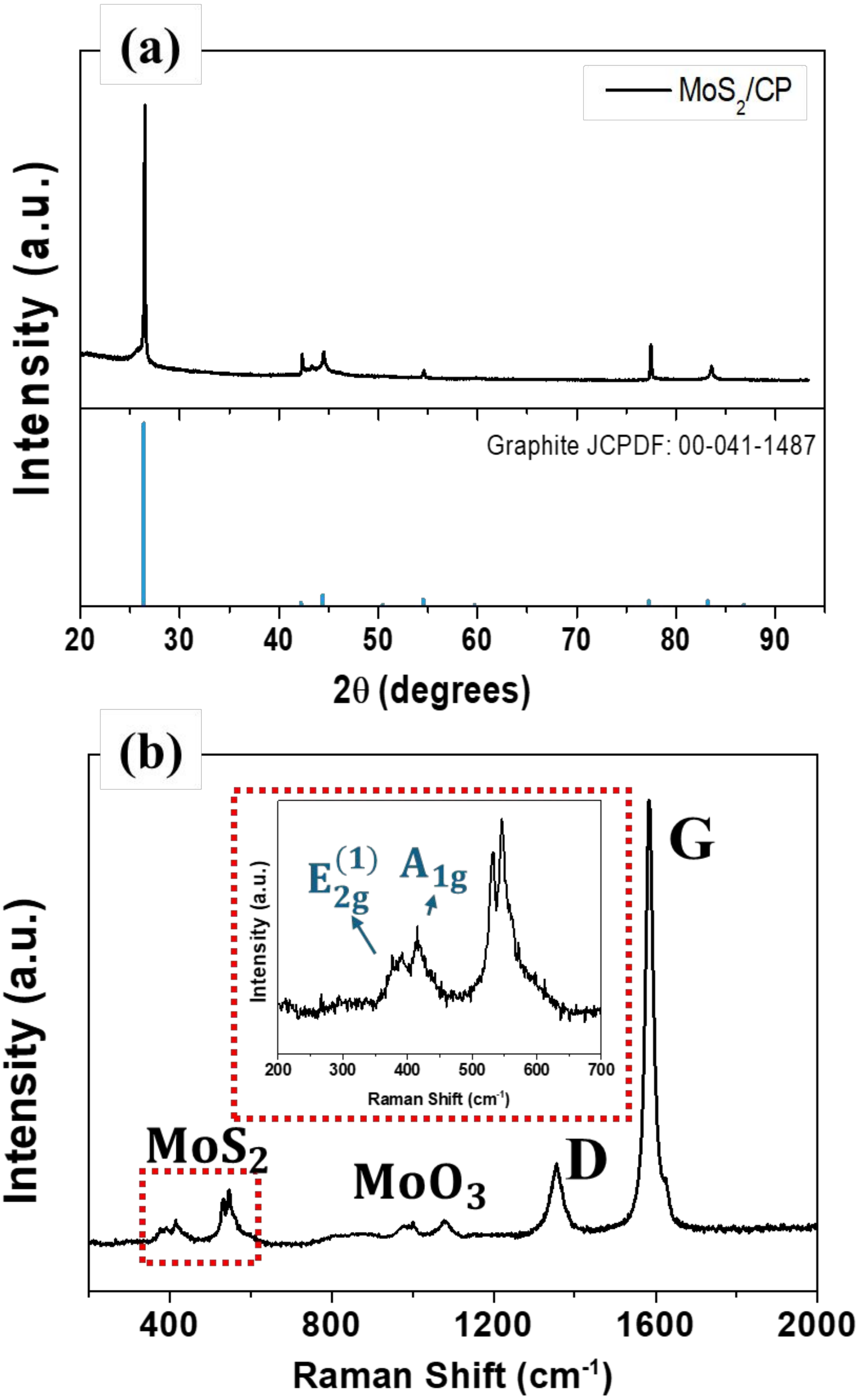

Figure S5. (a) XRD patterns for MoS_2/CP and (b) Raman spectra (532 nm) recorded on carbon paper and MoS_2/CP materials. Insert: Magnification of the region between 200 and 700 cm^{-1}, highlighting the vibration modes of MoS_2.

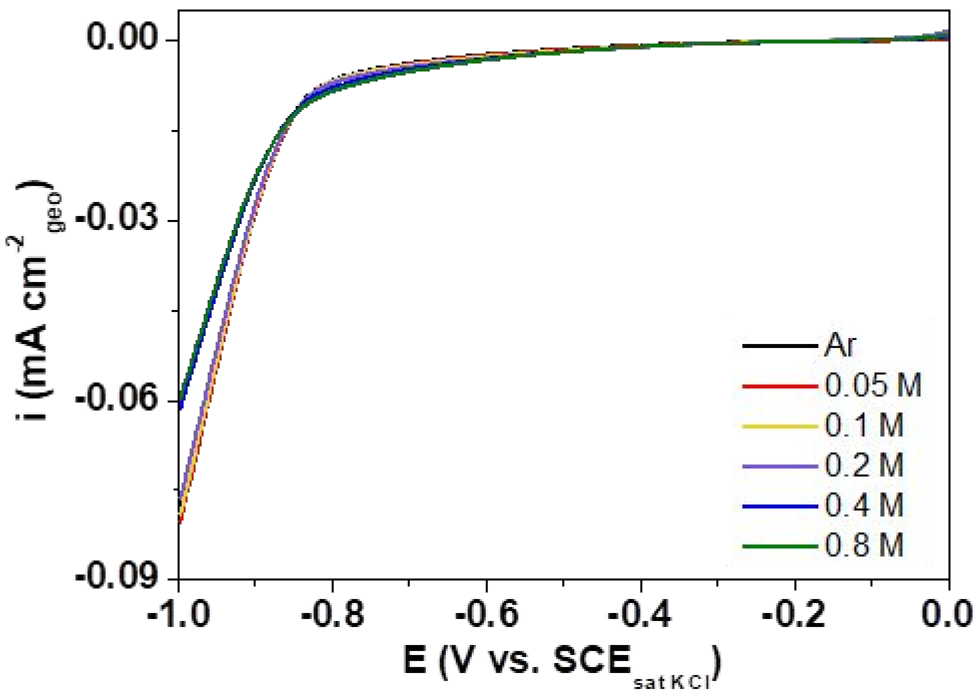

Figure S6. Linear sweep voltammograms (scan rate of 5 mV s^{-1}) for MoS_2/CP with different concentrations of acetone (0.05, 0.1, 0.2, 0.4 and 0.8 M) in 0.1 mol L^{-1} PBS (pH 7) electrolyte saturated with Ar.

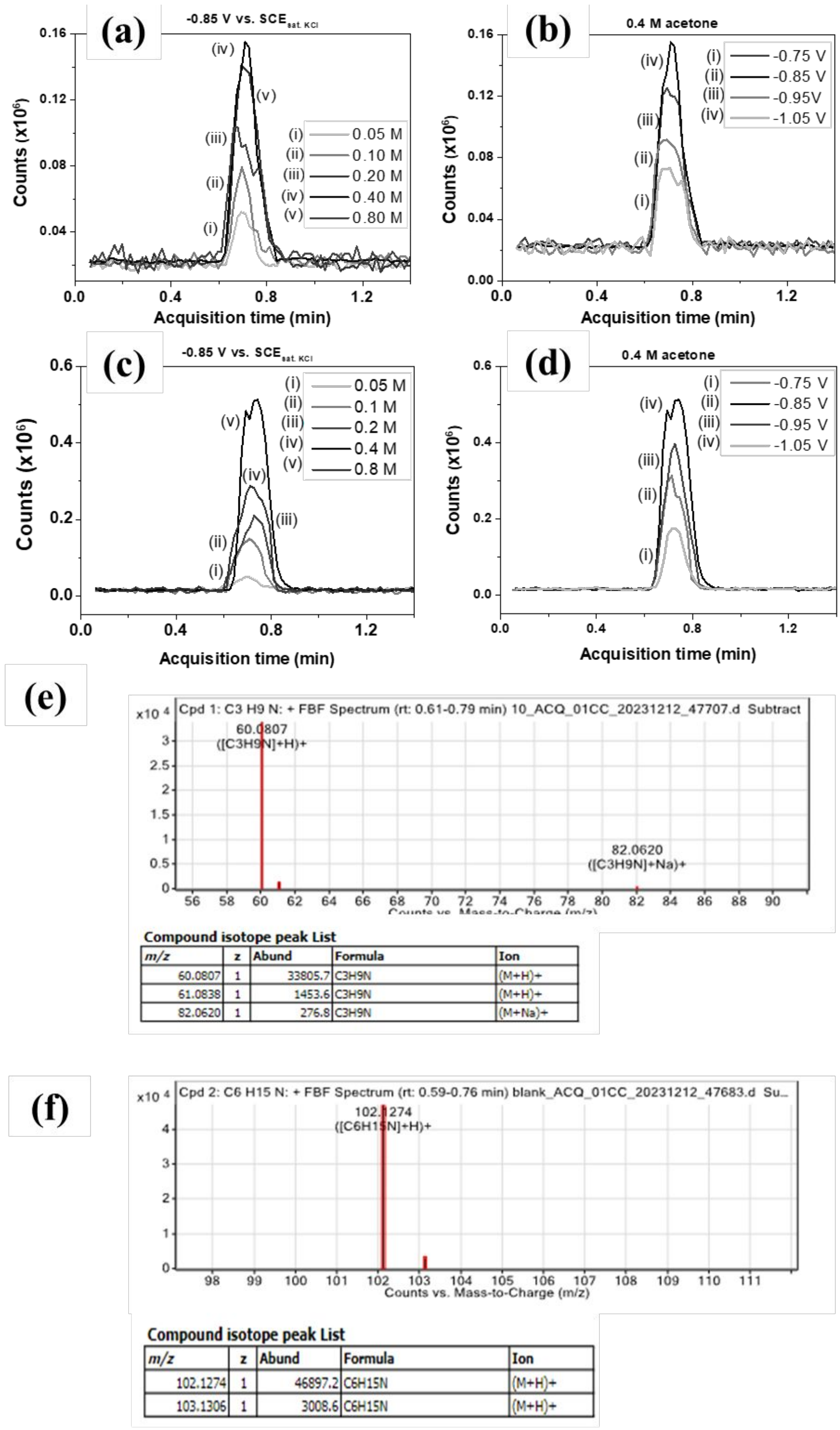

Figure S7. Corresponding counts versus acquisition time for LC-MS data for C_3H_9N (a, b) and $C_6H_{15}N$ (c, d) with different concentrations of acetone (0.05, 0.1, 0.2, 0.4 and 0.8 M) at –0.85 V vs. $SCE_{sat.\ KCl}$ (a,c) and (b,d) in 0.4 M acetone at different potentials. Identification of (e) isopropylamine (C_3H_9N) and (f) diisopropylamine ($C_6H_{15}N$) by mass spectroscopy (ion source: dual ESI). The peak of isopropylamine and diisopropylamine were identified by searching the possible m/z values of C_3H_9N and $C_6H_{15}N$, respectively, in cation mode, such as (M+H)+, (M+H)+ -H_2O and (M+Na)+.

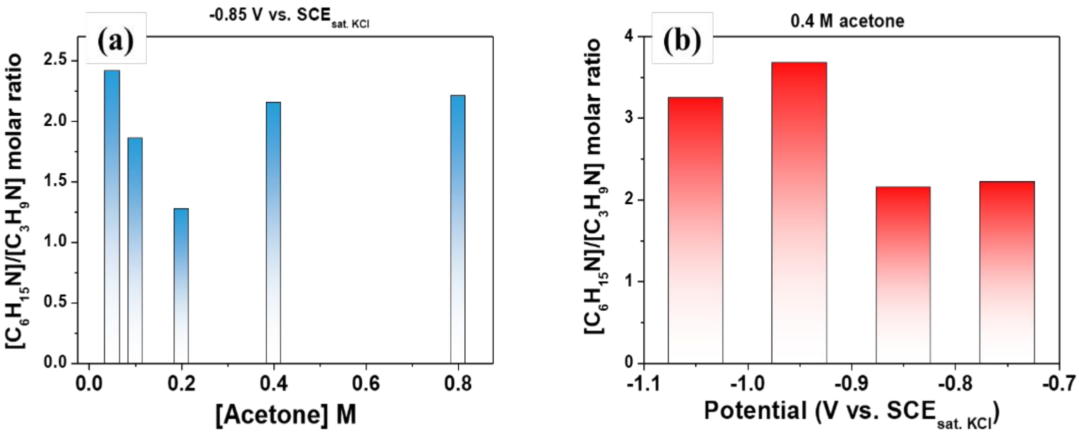

Figure S8. $[C_6H_{15}N]/[C_3H_9N]$ molar ratio (a) in different concentrations of acetone (0.05, 0.1, 0.2, 0.4 and 0.8 M) at –0.85 V vs. $SCE_{sat.\ KCl}$ and (b) in 0.4 M acetone at different potential applied. These values were taken from Figure 3c and Figure 3d, respectively.

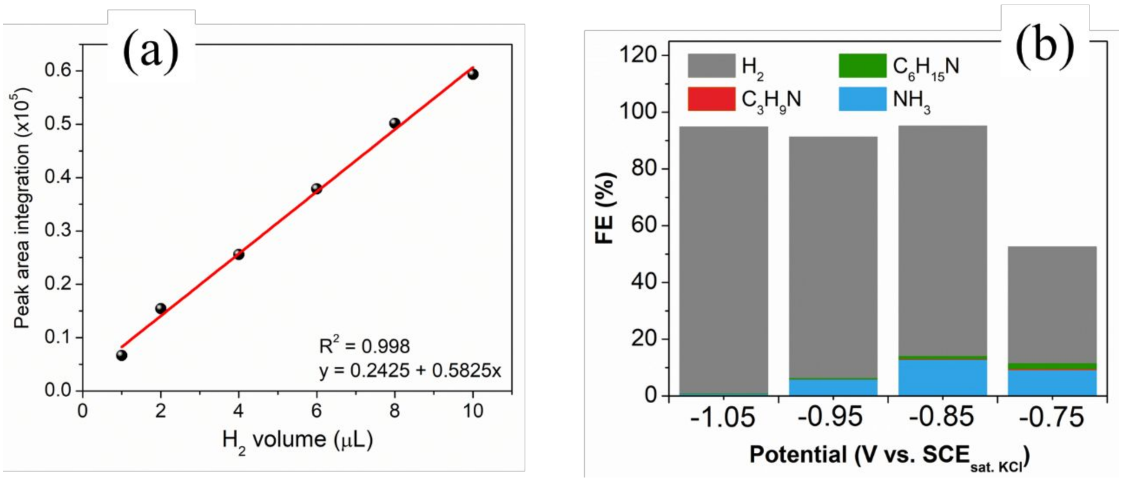

Figure S9. (a) The calibration curve used for calculation of H_2 volume. (b) The calculated FEs of H_2, NH_3, C3H9N and $C_6H_{15}N$ formation at various potentials with 0.4 M acetone.

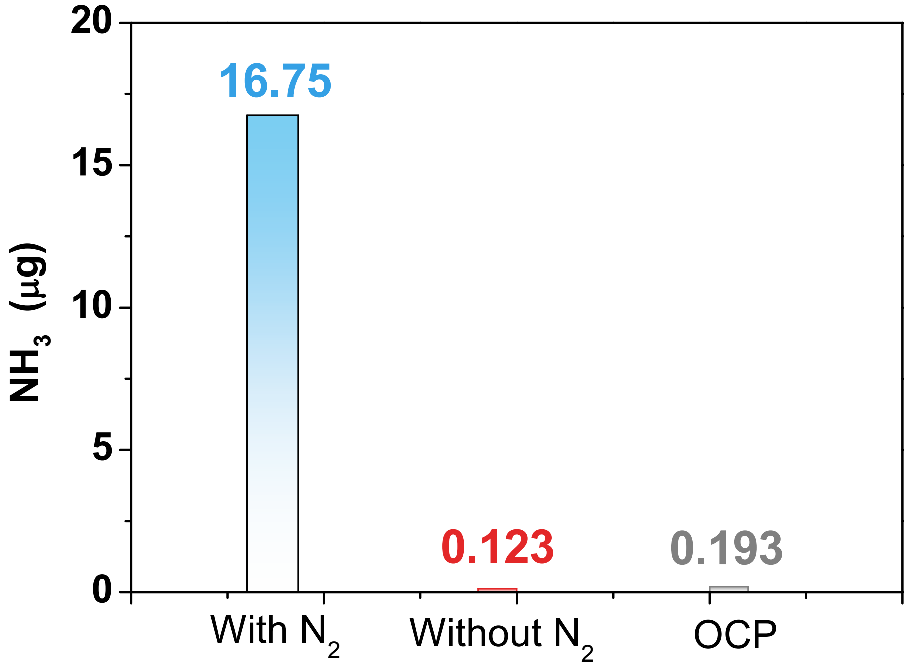

Figure S10. Control experiments carried out under different conditions: (i) N_2-saturated 0.1 M PBS + 0.4 M acetone electrolyte (ii) Ar-saturated 0.1 M PBS + 0.4 M acetone, and (iii) N_2-saturated electrolyte in an open circuit (OCP) condition.

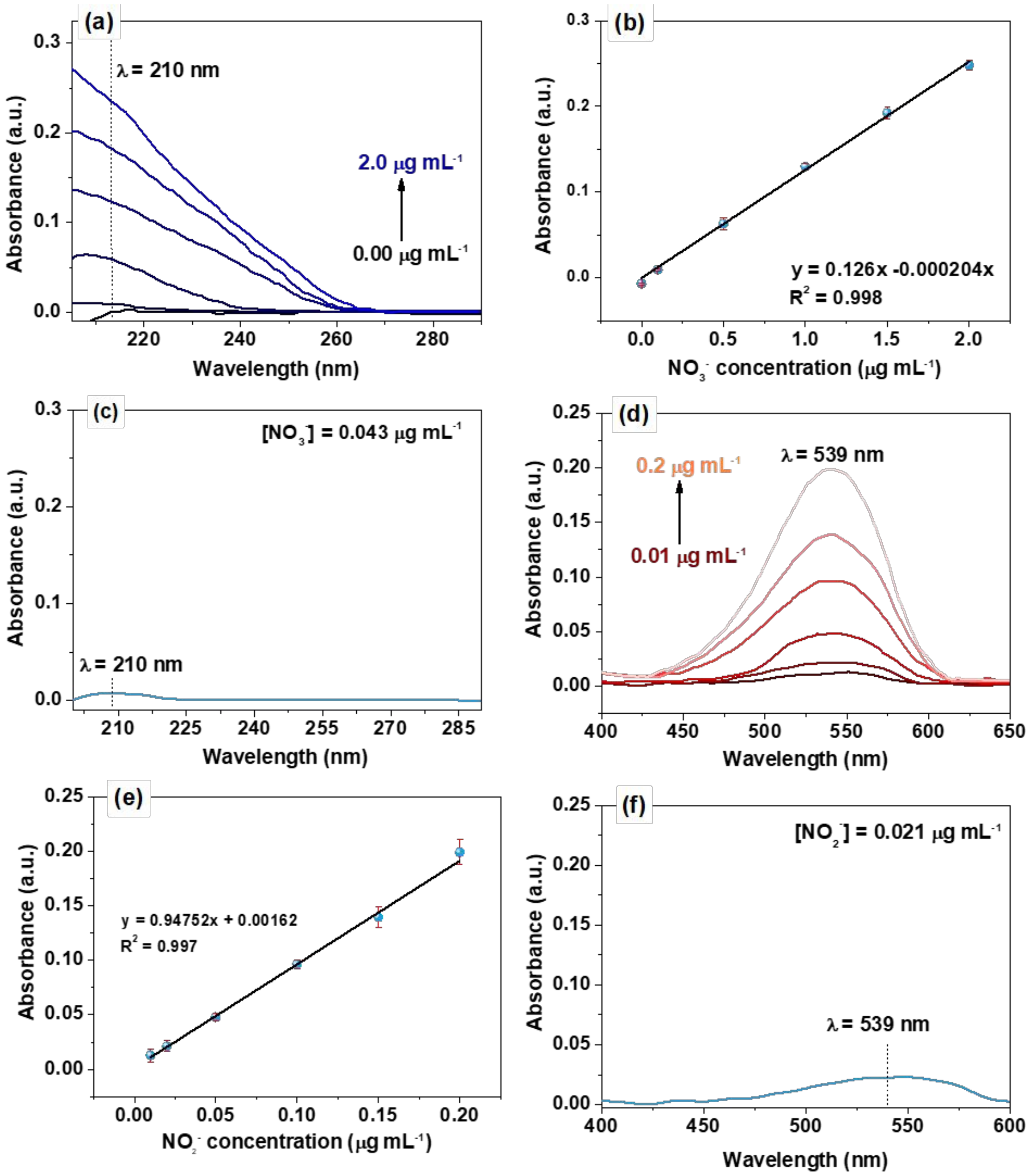

Figure S11. UV-Vis adsorption spectra of known concentration (0.00-2.0 μg mL^{-1}) of NO_3^- in 0.1 M Na_2SO_4. The absorbance at 210 nm was measured by UV-Vis spectrophotometer. (b) The corresponding calibration curve. (c) UV-vis adsorption spectrum of NO_3^- in N_2-saturated 0.1 mol L^{-1} PBS. (a) UV-Vis adsorption spectra of known concentration (0.05-2.0 μg mL^{-1}) of NO_3^- in 0.1 M PBS + 0.4 M acetone. The absorbance at 539 nm was measured by UV-Vis spectrophotometer. (b) The corresponding calibration curve. (c) UV-vis adsorption spectrum of NO_2^- in N_2-saturated 0.1 mol L^{-1} PBS + 0.4 M acetone.

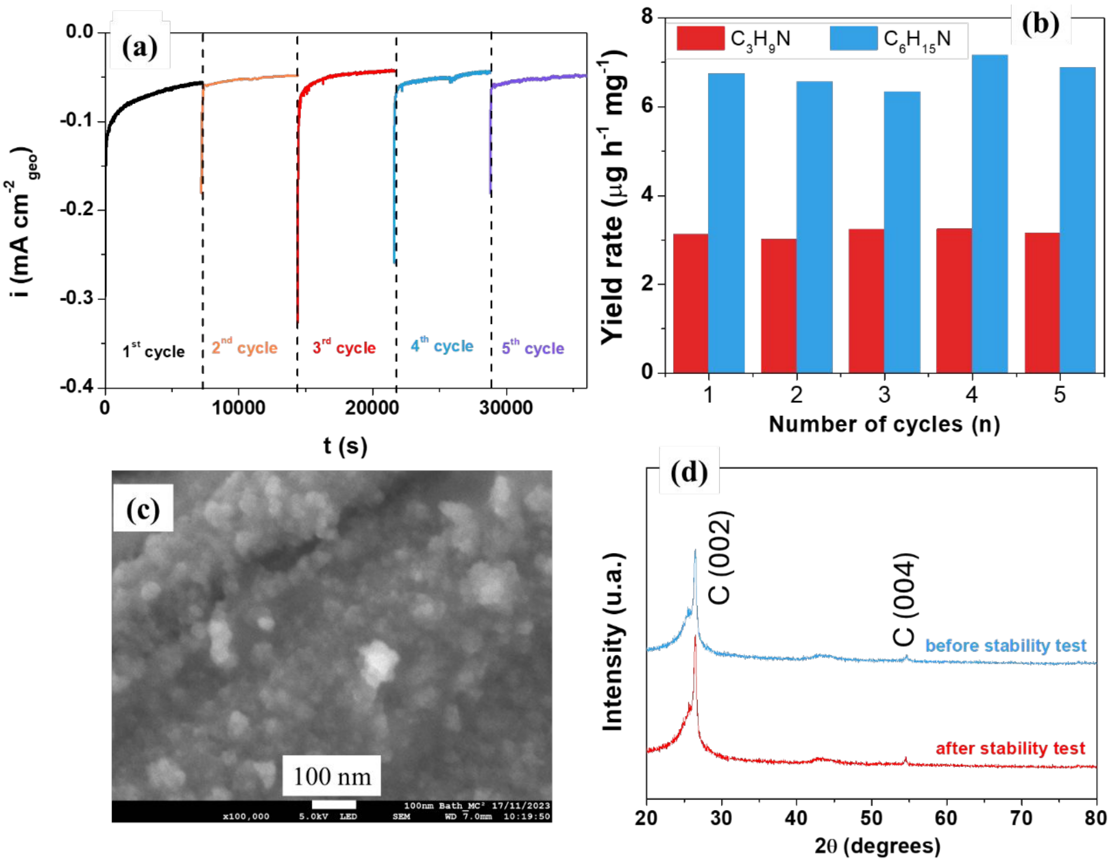

Figure S12. (a) time-dependent current density curves. (b) C_3H_9N and $C_6H_{15}N$ yield rates for MoS_2/CP at –0.85 V vs. $SCE_{sat.\ KCl}$ in different cycles of NRR + 0.4 M acetone. (c) SEM image after the cycling stability test. (d) X-ray diffraction patterns for MoS_2/CP catalyst before and after cycling stability test.
